# One Swallow Does Not Make a Summer: A Confirmed Case of Imported *Vibrio cholerae* After *Clostridioides difficile* Diagnosis in Brescia, Northern Italy

**DOI:** 10.1155/crdi/5552486

**Published:** 2026-01-14

**Authors:** Cristina Seguiti, Elia Croce, Enza Granato, Alessia Giovannini, Stefano Grossi, Marcello Cecere, Daniela Fortini, Laura Villa, Anna Caruana, Gabriele Del Castillo, Luigi Vezzosi, Danilo Cereda, Angelo Meloni, Paolo Colombini

**Affiliations:** ^1^ Infectious Diseases Unit, Fondazione Poliambulanza Istituto Ospedaliero, Brescia, Italy, poliambulanza.it; ^2^ Internal Medicine and Geriatrics, Fondazione Poliambulanza Istituto Ospedaliero, Brescia, Italy, poliambulanza.it; ^3^ Healthcare Department, Fondazione Poliambulanza Istituto Ospedaliero, Brescia, Italy, poliambulanza.it; ^4^ Intensive Care Unit, Fondazione Poliambulanza Istituto Ospedaliero, Brescia, Italy, poliambulanza.it; ^5^ Department of Laboratory Medicine, Fondazione Poliambulanza Istituto Ospedaliero, Brescia, Italy, poliambulanza.it; ^6^ Internal Medicine Department, Fondazione Policlinico Universitario A. Gemelli IRCCS-UCSC, Rome, Italy; ^7^ Department of Infectious Diseases, Istituto Superiore di Sanità, Rome, Italy, iss.it; ^8^ Department of Hygiene and Prevention, Infectious Diseases Unit, ATS, Brescia, Italy; ^9^ Infectious Diseases and Vaccinations, Direzione Generale Welfare, Regione Lombardia, Milan, Italy; ^10^ Department of Prevention, Direzione Generale Welfare, Regione Lombardia, Milan, Italy

**Keywords:** *Clostridioides difficile*, coinfection, *Vibrio cholerae*

## Abstract

*Vibrio cholerae*, the aetiological agent of cholera, is predominantly an imported pathogen in high‐income countries. We report a case of a 49‐year‐old Nigerian male returning from Africa with abdominal pain and watery diarrhea, who rapidly developed acute kidney injury. The initial diagnosis was *Clostridioides difficile* enteritis, based on GDH antigen and toxin detection in stool samples. Microbiological cultures subsequently revealed concomitant growth of *Vibrio cholerae*. The patient required intensive care unit management, including aggressive fluid resuscitation and antimicrobial treatment with doxycycline and vancomycin. Surveillance cultures were performed on both patients and patients’ contacts; no additional cholera cases were detected during follow‐up. This case highlights the importance of considering cholera in patients presenting with diarrheal syndromes after returning from endemic regions, even when another, more common pathogen in high‐income countries has already been identified. Coinfection may worsen clinical outcomes and has significant implications for both therapeutic decisions and infection control measures.

## 1. Introduction

*Vibrio cholerae* is a Gram‐negative bacterium recognized as the aetiological agent for cholera, a watery diarrheal syndrome transmitted via the faecal–oral route through contaminated water and food. Based on the O antigen of its lipopolysaccharide, *V. cholerae* is classified into multiple serogroups, among which O1 and O139 are responsible for epidemic outbreaks [1]. Cholera toxin induces watery diarrhoea by stimulating adenylate cyclase activity in intestinal epithelial cells, leading to the secretion of large volumes of ion‐rich fluid that exceed the absorptive capacity of the intestine [2].

Cholera remains a major cause of morbidity in low‐income countries, where inadequate sanitization facilitates large outbreaks. However, the true burden of the disease is frequently underestimated due to underreporting [3]. In an increasingly globalized context, it is essential for clinicians to maintain a high index of suspicion for imported infections when evaluating patients with relevant epidemiological risk factors. Particular attention should be paid to the possibility of coinfections with pathogens that are more prevalent in high‐income settings, as these may obscure the primary diagnosis.

## 2. Case Report

In January 2025, a 49‐year‐old man who had recently returned to Brescia, Northern Italy, from a trip to his homeland, Nigeria, presented to our emergency department (ED) with a one‐day history of vomiting and abdominal pain. Mild symptoms had started during his flight. While in the ED, his condition rapidly deteriorated, with worsening abdominal pain accompanied by profuse watery diarrhea. Laboratory investigations documented leukocytosis and a mild elevation of C‐reactive protein. A stool sample tested positive for toxigenic *Clostridioides difficile*, and treatment with oral vancomycin (125 mg every six hours for 10 days) was initiated alongside isolation precautions.

**Figure 1:**
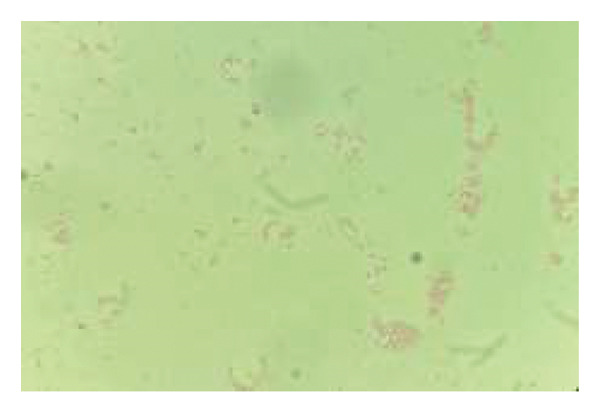
Identification of Gram‐negative bacilli by optical microscopy (Gram stain).

**Figure 2:**
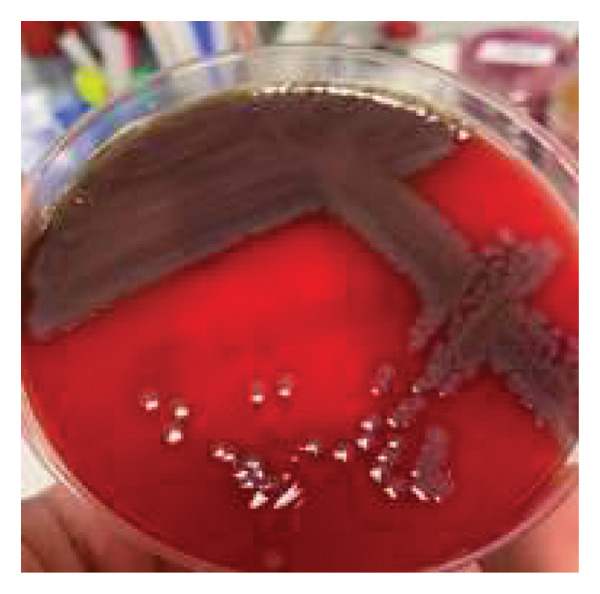
*Vibrio cholerae* colonies on blood agar base.

Within a few hours, the patient developed acute kidney injury (serum creatinine: 8.38 mg/dL), hypotension, and metabolic acidosis. Due to progressive clinical deterioration, the patient was transferred to the intensive care unit (ICU) for aggressive fluid resuscitation and hemodynamic stabilization. Stool cultures subsequently confirmed an infection with *Vibrio cholerae*, initially identified by MALDI‐TOF mass spectrometry and colony morphology on agar plate (Figures 1 and 2), with an identification confidence of 99.9%. Confirmation was later obtained from the Italian National Institute of Health, which identified *V. cholerae* O1, serotype Ogawa, and Haitian cholera toxin variant. Notably, the patient was already under isolation precautions due to concurrent *C. difficile* infection.

A single 300 mg dose of doxycycline was administered in accordance with CDC guidelines, pending antimicrobial susceptibility results. Susceptibility testing, performed with the Vitek 2 system (bioMérieux), demonstrated resistance to macrolides but susceptibility to tetracycline and quinolones. The case was promptly reported to the Regional Health Authority, and the patient was interviewed to enable contact tracing and follow‐up measures.

During the first 24 h, the patient experienced an estimated fluid loss of 6 L through diarrhea. An aggressive rehydration protocol, consistent with CDC recommendations for cholera management, was implemented: intravenous Ringer′s lactate at 30 mL/kg of Ringer′s lactate over the first 30 min, followed by 70 mL/kg over the subsequent 3 h, together with electrolytes imbalance correction. Once clinically stable and able to tolerate oral intake, he was transitioned to oral rehydration solution for maintenance therapy. The patient showed progressive improvement with normalization of renal function over the following days. He was discharged from the ICU after 4 days and transferred to the general medicine ward under continued isolation and clinical monitoring to complete a 10‐day treatment course for associated *C. difficile* infection. After documentation of three consecutive negative stool cultures and PCR tests for *V. cholerae*, the patient was discharged home and reintegrated into the community. No secondary cases of cholera were identified among his close contacts.

## 3. Discussion

According to the European Centre for Disease Prevention and Control (ECDC), a total of 490.700 cholera cases, including 3.693 deaths, were reported worldwide between 1 January 2024 and 25 November 2024. The five countries reporting the highest case counts were Afghanistan, Sudan, Democratic Republic of the Congo, Tanzania, and Burundi [4]. Despite the global burden, cholera remains rare in Europe and is almost exclusively associated with travel to endemic areas. The most recent ECDC Report documented 29 imported cases in Europe in 2022; this is the highest number recorded since 2011 [5]. Accordingly, cholera should be strongly suspected in a patient with compatible symptoms and a relevant epidemiological link, such as travel to endemic countries. In our case, the patient had returned from Nigeria, where he had visited friends and relatives (VFR travel).

Between 2018 and 2023, only two cases of cholera were reported in Italy, both associated with either travel to endemic areas or consumption of food imported from Bangladesh [6, 7]. This underscores not only the rarity of the disease in high‐income countries but also highlights the importance of awareness among clinicians.

The cornerstone of cholera management remains prompt and aggressive fluid resuscitation, while antibiotic therapy serves to reduce both symptom duration and pathogen shedding. Coinfections with gastrointestinal pathogens other than *C. difficile* have been described in the literature, including *Campylobacter* spp., *Escherichia coli*, and *Rotavirus* [6]. Coinfecting microorganisms may modulate the gene expression of the primary pathogen, potentially enhancing its virulence, worsening clinical outcomes, disease severity, and increasing healthcare‐related costs. This phenomenon has been explored by the work of Abdel‐Haleem et al. on genome‐scale modelling of *V. cholerae* and enterotoxigenic *E. coli* (ETEC) [8]. To the best of our knowledge, this is the first reported case of imported cholera occurring in association with *C. difficile* infection.

## 4. Conclusion

Cholera should be considered as a potential diagnosis in travelers returning from endemic areas, particularly in cases presenting with profuse watery diarrhea, even when another microbial pathogen has already been identified. The possibility of concurrent gastrointestinal infections underscores the need for thorough microbiological evaluation, as coinfections may delay the initiation of appropriate treatment, adversely affect clinical outcomes, and complicate infection control strategies.

## Ethics Statement

The preparation of this case report was conducted in accordance with the international standards and ethical guidelines for case reporting. The patient was informed about the potential publication of this case and provided consent. Full anonymization was ensured at every stage of manuscript preparation, in compliance with the National Scientific Guidelines on Case Reports.

## Consent

Please see the Ethics Statement.

## Conflicts of Interest

The authors declare no conflicts of interest.

## Funding

No funding was received for this study.

## Supporting Information

This case report has been prepared in accordance with the CARE (CAse REport) guidelines, and the CARE checklist is provided as a supporting information (CARE checklist.pdf).

## Supporting information


**Supporting Information** Additional supporting information can be found online in the Supporting Information section.

## Data Availability

The data that support the findings of this study are available from the corresponding author upon reasonable request.
